# Identification of the Target for a Transition Metal-α-Amino Acid Complex Antibiotic Against *Mycobacterium smegmatis*

**DOI:** 10.3389/fphar.2021.686358

**Published:** 2021-06-25

**Authors:** George W. Karpin, Joseph S. Merola, Joseph O. Falkinham

**Affiliations:** ^1^ Virginia Tech Center for Drug Discovery and Development, Blacksburg, VA, United States; ^2^ Department of Chemistry, Virginia Tech, Blacksburg, VA, United States; ^3^ Department of Biological Sciences, Virginia Tech, Blacksburg, VA, United States

**Keywords:** transition metal-α-amino acid complexes, mycobacteria, clarithromycin, 23S rRNA, peptidyl transferase

## Abstract

Spontaneous mutants of *Mycobacterium smegmatis* strain mc^2^155 resistant to **1-PG** (iridium-L-phenylglycine complex), an antimycobacterial antibiotic, were isolated. Based on the discovery that some **1-PG**-resistant mutants (**1-PG** ^**R**^) were also resistant to high concentrations of clarithromycin (≥250 μg/ml), but no other anti-mycobacterial antibiotics, the 23S rRNA region spanning the peptidyl transferase domain was sequenced and mutations shown to be localized in the peptidyl transferase domain of the 23S rRNA gene. Measurements showed that **1-PG** bound to ribosomes isolated from the **1-PG**-sensitive parental strain, but the ribosome binding values for the **1-PG** ^**R**^ mutant reduced.

## Introduction

There is a well established recognition that new antibiotics for the treatment of mycobacterial infections are needed (Raviglione and Ditiu, 2013; Zumla et al., 2013). Further, the prevalence of mycobacterial pulmonary diseases caused by the nontuberculous mycobacteria (e.g., *Mycobacterium avium* complex, MAC) is estimated at 10–15 cases per 100,000 individuals and increasing at a rate of 5–8% per year in the United States and Canada (Marras et al., 2007; Billinger et al., 2009; Winthrop et al., 2010). In addition, as NTM-infected individuals are susceptible to re-activation or re-infection after anti-mycobacterial therapy (Wallace et al., 2002; Wallace et al., 2014; Min et al., 2015). In individuals over 60 years NTM-pulmonary disease prevalence approaches 100 cases per 100,000 (Prevots et al., 2010). It would follow that as the proportion of the United States population over 60 years increases to 25% by 2025, it is likely that the prevalence of nontuberculous mycobacteria (NTM) disease will continue to increase, as will the need for novel anti-mycobacterial drugs.

Recently we have described the synthesis and antimycobacterial activity of transition metal-α-amino acid complexes (Karpin et al., 2013). The transition metals, iridium (Ir), ruthenium (Ru), rhodium (Ro) have been complexed with a variety of α-amino acids. Of the family of complexes synthesized, Ir-phenylglycine (**1-PG**) exhibited anti-mycobacterial activity with MICs of 5 μg/ml against *Mycobacterium smegmatis*, 31 μg/ml against *Mycobacterium abscessus*, and 15 μg/ml against *Mycobacterium intracellulare, Mycobacterium chelonae*, and *Mycobacterium bovis* BCG (Karpin et al., 2013). As **1-PG** was shown to lack cytotoxic and hemolytic activities (Karpin et al., 2013), it was decided to proceed with the identification of the drug’s target.

Herein we report the isolation of **1-PG**-resistant mutants of *M. smegmatis* and the identification of at least one target of the drug, the peptidyl transferase domain of the mycobacterial 23S rRNA.

## Materials and Methods

### Chemistry

**FIGURE 1:**
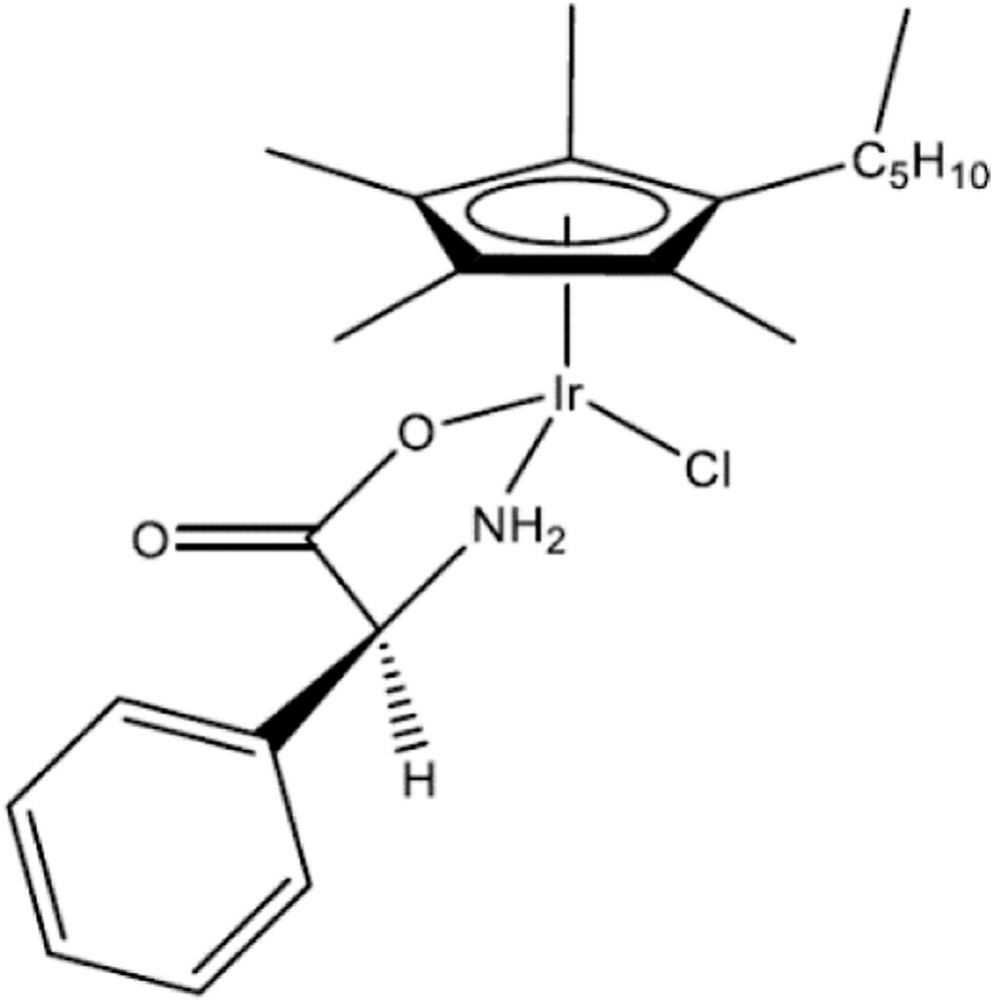
Structure of **1-PG**. (η^5^-pentamethylcyclopentadienyl) (phenylglycine) chloroiridium.

The structure of the transition metal-α-amino acid complex **1-PG** is illustrated in Figure 1. **1-PG** is a cyclopentadienyl (Cp*) having an α-amino acid, here phenylglycine, complexed with iridium (Ir). **1-PG** synthesis has been described (Karpin et al., 2013).

### Antimycobacterial Antibiotics

The antimycobacterial antibiotics, clarithromycin, ethambutol, ciprofloxacin, and were purchased from Sigma-Chemical Co. (St. Louis, MO)

### *Mycobacterium smegmatis* and Growth

*M. smegmatis* strain mc^2^155*,* was used in the study and its growth and preparation for susceptibility measurements are described in Falkinham et al. (Falkinham et al., 2012). Cultures were grown to mid-logarithmic phase to obtain uniformity of MIC measurements.

### Measurement of Minimal Inhibitory Concentrations (MIC) and Minimal Bactericidal Concentrations (MBC)

MICs and MBCs of compounds dissolved in M7H9 broth medium containing 0.5% (vol/vol) glycerol and 10% (vol/vol) oleic acid-albumin were measured by broth microdilution in 96-well microtitre plates (Falkinham et al., 2012).

### Selection of 1-PG-Resistant (1-PG^R^) Mutants

Samples (0.1 ml) of a stationary phase culture of *M. smegmatis* strain mc^2^155 were spread on M7H10 agar medium containing 10% (vol/vol) oleic acid-albumin and concentrations of **1-PG** ranging from 4–20 µg **1-PG**/ml. Plates were incubated at 37°C and single colonies picked from the plate containing 20 µg 1_PG/mL and streaked for purification on both **1-PG**-containing and **1-PG**-free medium (to exclude **1-PG**-dependant mutants). Following isolation, the MICs of the parent and mutants were measured against **1-PG** and other antimycobacterial antibiotics as described above.

### Isolation of DNA, PCR Amplification and Sequencing a Portion of the 23S rRNA Gene

DNA was isolated from the parent and mutant A1 **1-PG** ^**R**^ mutant and the 23S rRNA gene was amplified by PCR as described (Meier et al., 1994; Jamal et al., 2000), resulting in production of 419 bp amplicon (domain V) of the 23S rRNA gene). The 419 bp amplicon was sequenced (Sanger) at the Virginia Tech Biocomplexity Institute employing an ABI 3730 (Applied Biosystems).

### Isolation of *M. smegmatis* Ribosomes

Ribosomes were isolated from *M. smegmatis* strain mc^2^155 and the **1-PG** ^R^ mutants following the procedure of Doucet-Populaire et al. (Doucet-Populaire et al., 1998). Cells were harvested from 50 ml cultures by centrifugation (5,000 × *g* for 20 min), supernatant medium discarded, and cells washed twice in 50 ml of Buffer A (10 mM Tris-HCl, 4 mM MgCl_2_, 10 mM NH_4_Cl, 100 mM KCl, pH 7.2). Washed cells were suspended in 5 ml of Buffer A, cell suspensions were cooled on ice-water, and cells lyzed by sonication. DNase (RNase-free) was added to the cooled and broken cell suspensions at a final concentration of 5 units/ml and incubated on ice for 15 min. Whole cells were removed from the lysate by centrifugation (5,000 × *g* for 5 min) and the supernatant transferred to an ultracentrifuge tube and centrifuged at 30,000 × *g* for 30 min to pellet cell walls and membranes. The supernatant from that centrifugation was transferred to a fresh ultracentrifuge tube and ribosomes pelleted at 100,000 × *g* for 60 min. The pelleted ribosomes were suspended in 2 ml of Buffer A, aliquot in 0.5 ml samples, labeled and frozen at −70°C.

### Measurement of 1-PG-Binding to Ribosomes

**1-PG**-binding to ribosomes was measured as described by Douthwait and Aagaard (Douthwaite and Aagaard, 1993). An aliquot of each strain’s (i.e., mc^2^155 and **1-PG** ^**R**^ mutants) ribosome suspension was defrosted and 50 µl of ribosomes was mixed with 50 µl of 1 mg **1-PG**/ml and incubated at 37°C. Immediately and at 10 min intervals up to 30 min, two 10 µl samples were withdrawn, filtered through 0.45 µm pore size filters, and washed with 5 ml of Buffer A. The filters were placed in a tube, 1 ml of 1 M HMO_3_ added, and the concentration of Ir measured by Inductively Coupled Plasma—Optical Emission Spectroscopy (ICP-OES).

### Inductively Coupled Plasma—Optical Emission Spectroscopy Measurements of Ir in Digested Ribosome Fractions

The HNO_3_-digests were diluted with Nanopure® deionized water to a final volume of 5 ml. Each sample was allowed to sit for 15 min at room temperature. Iridium in samples was measured as all iridium isotopes using a Perkin Elmer 4300 DV ICP-OES. Calibration was performed following the manufacturer’s directions. Iridium in both the filters and the filtrates were measured independently to determine what may have been bound to the ribosome and/or washed through the filter paper containing the free complex. A standard curve was constructed using samples of iridium prepared for ICP-OES by Inorganic Ventures (Christiansburg, VA).

## Results and Discussion

### Selection of 1-PG-Resistant (1-PG^R^) Mutants

Seven (7) mutants of *M. smegmatis* strain mc^2^155 from a single culture resistant to 20 µg **1-PG**/ml) were isolated (frequency = 3.5 × 10^–7^) and their susceptibility to anti-mycobacterial antibiotics measured (Table 1). MIC increases of the **1-PG** ^**R**^ mutants was modest compared to their **1-PG** ^S^ parent strain mc^2^155 and two, namely mutants D1 and F1, had MICs equal to that of the **1-PG** ^**S**^ parent (Table 1). All seven were also resistant to clarithromycin (MIC = 2–16-fold higher than parent), but their susceptibilities to other anti-mycobacterial drugs were not different from that of the parent strain (Table 1). Based on the fact that mutants A1 and B1 retained the dry colony morphology of the parent and did not produce mucoid colonies as did mutants B2, C1, and E1, mutant strain A1 was investigated further.

**TABLE 1:** MICs of Standard Mycobacterial Drugs Against 1-PG-resistant Mutants of *Mycobacterium smegmatis* strain mc^2^ 155. The bold values are short form name of the antimycobacterial compound in Figure 1, **1-PG.**

Antibiotic	Minimal inhibitory concentration (MIC) in µg/mL of strains							
	mc^2^a155	A1	B1	B2	C1	D1	E1	F1
**1-PG**	2	8	8	8	16	2	16	2
Clarithromycin	32	>250	250	125	62.5	125	62.5	125
Rifampin	125	250	125	125	250	125	250	125
Ethambutol	2	1	1	2	2	1	2	1
Ciprofloxacin	0.25	0.25	0.25	0.25	0.25	0.25	0.25	0.25
Streptomycin	0.5	0.25	0.5	0.5	0.5	0.5	1	0.5
Isoniazid	25	12.5	25	12.5	12.5	25	25	25

### Isolation of DNA, PCR Amplification, and Sequence of a Portion of the 23S rRNA Gene

As clarithromycin-resistant mutants have mutations in the peptidyl transferase domain (V) of the 23S rRNA gene (Meier et al., 1994; Jamal et al., 2000), DNA was isolated from the parent and mutant A1 and the 23S rRNA gene was amplified by PCR [12.13], resulting in production of 419 bp amplicon (domain V). Analysis of the sequence of the 419 bp amplicon in the **1-PG** ^R^/Cla^R^ mutant revealed a substitution of T for G at position 79 (base 2057 of the 23S rRNA) and a substitution of C for a T at position 120 (base 2611of the 23S rRNA gene) (Table 2). Both are within the peptidyl transferase loop (domain V) of the 23S rRNA gene. As there were no other base changes in the **1-PG** ^**R**^-mutant, we surmise that those mutations rendered the *M. smegmatis* mutant strain resistant to **1-PG** and clarithromycin because of alterations in the conformation of that loop (Doucet-Populaire et al., 1998; Douthwaite and Aagaard, 1993).

**TABLE 2:** Sequence analysis of Cla^S^ (parent) and Cla^R^ mutant *M. smegmatis* strains.

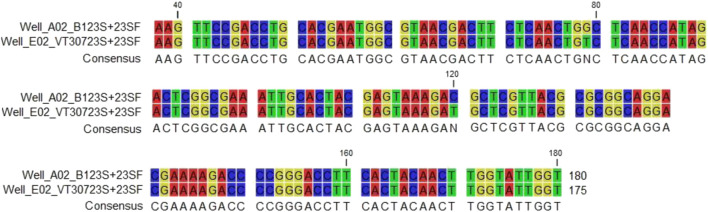

### Binding of 1-PG to Ribosomes

The concentrations of total iridium (ppm) in the filtrate and filter-bound ribosomes of the **1-PG** ^**R**^ mutant strain A1 and its parent are shown in Table 3. Each value in the table is an average of five readings and the results of two independent Ir-binding studies are listed (Experiments 1 and 2). The ribosome fractions from the **1-PG** ^**R**^-strain bound less iridium than those of the parent **1-PG** ^**S**^-strain. (Table 2). Further, more iridium (as **1-PG**) was recovered in the filtrate of the **1-PG** ^**R**^-strain than from the filtrate of the **1-PG** ^**S**^-parental strain which is in agreement with the hypothesis that a possible alteration in the structure of the peptidyl transferase site of the ribosomal fraction led to reduced **1-PG**-binding and resistance.

**TABLE 3:** Iridium-binding of filter-bound ribosomes of **1-PG** ^**S**^ *M. smegmatis* parent and **1-PG** ^**R**^ mutant A1. The bold values are short form name of the antimycobacterial compound in Figure 1, **1-PG**.

Experiment	Iridium (ppm)/µg/RNA ^a^	
Strain	Filter-bound ribosomes	Filtrate
Experiment 1		
**1-PG** ^**S**^ parent	1.2 ± 0.1	0.6 ± 0.04
**1-PG** ^**R**^ mutant	0.5 ± 0.05	1.5 ± 0.1
Experiment 2		
**1-PG** ^**S**^ parent	1.6 ± 0.04	>0.5
**1-PG** ^**R**^ mutant	0.6 ± 0.005	1.1 ± 0.14

a Average ± standard deviation of five measurements.

### Isolation and Characterization of Clarithromycin-Resistant Mutants

Independent Cla^R^-mutants of mc^2^155 were also isolated, but only 4/7 were **1-PG** ^**R**^, suggesting the two antibiotics do not share exactly the same range of activity. This separation of targets is consistent with observations that clarithromycin inhibits peptidyl transferase activity, ribosome assembly, and outer membrane assembly in mycobacteria (Doucet-Populaire et al., 1998). Support for that contention could be obtained by demonstration of co-transduction of resistance to both antibiotics (Lee et al., 2004). The discovery that a substantial fraction (43%) of clarithromycin-resistant *M. smegmatis* mutants (Cla^R^) were still susceptible to Ir-phenylglycine (**1-PG** ^**S**^) encourages us that **1-PG** will prove to be a useful anti-mycobacterial drug, even in infections due to a Cla^R^ *Mycobacterium* spp. strain.

## Data Availability

The datasets presented in this study can be found in online repositories. The names of the repository/repositories and accession number(s) can be found in the article/Supplementary Material.
